# Draft Whole-Genome Sequence of Bacillus paramycoides LB_RP2, a Putative Polyhydroxyalkanoate-Producing Bacterium Isolated from an Amazonian Blackwater River

**DOI:** 10.1128/MRA.00438-21

**Published:** 2021-07-29

**Authors:** Lorena Mota de Castro, Choon Pin Foong, Mieko Higuchi-Takeuchi, Eraldo Ferreira Lopes, Keiji Numata, Suelen Dias da Silva, Luana da Silva Nonato, Adolfo José da Mota, José Odair Pereira

**Affiliations:** a Biodegradation Laboratory, Bionorte Biotechnology Graduate Network, Universidade Federal do Amazonas (UFAM), Manaus, Amazonas, Brazil; b Department of Material Chemistry, Graduate School of Engineering, Kyoto University, Nishikyo-ku, Kyoto, Japan; c Biomacromolecules Research Team, RIKEN Center for Sustainable Resource Science, Hirosawa, Wako, Saitama, Japan; d Institute of Health and Biotechnology of Coari, Universidade Federal do Amazonas, Coari, Amazonas, Brazil; e National Institute for Amazonian Research, INPA, Manaus, Amazonas, Brazil; University of Rochester School of Medicine and Dentistry

## Abstract

Bacteria of the genus *Bacillus* have been investigated due to the ability that many species have of accumulating polyhydroxyalkanoates (PHA) via a wide variety of raw materials as their carbon source. Herein, we report the draft whole-genome sequence of the putative PHA-accumulating strain Bacillus paramycoides LB_RP2, isolated from an Amazonian river.

## ANNOUNCEMENT

Metabolic features such as genetic stability, a higher growth rate, and the capacity to use nonfeedstock substrates as energy and carbon sources turn *Bacillus* species into model organisms in the field of research. In addition, many of these species are being investigated due to their ability to accumulate polyhydroxyalkanoates (PHA) (1). Herein, we report the draft whole-genome sequence of Bacillus paramycoides strain LB_RP2.

The microorganism was isolated from surface water collected from the Preto River, located in Barcelos, Amazonas State, Brazil (0°32′28.5″N, 62°29′52.2″W). This strain was cultivated in lysogenic broth (LB) (Lennox) (5 ml/30°C/120 rpm), and cells were washed and plated onto agar (18 g/liter) plates with mineral saline (MS) modified medium (2) supplemented with 1 ml of trace element solution (3), 1.0 g/liter of glucose, and Nile red (0.5 μg/ml). After 48 h of incubation (30°C), the plates were exposed to 302 nm UV light in a transilluminator (Kasvi), and a fluorescent colony identified as RP2 was selected. Gram stain and cell morphology were determined according to the manufacturer’s protocol (Laborclin kit; Brazil). The catalase activity was analyzed according to the standard method (4).

The isolate was grown on LB at 30°C and harvested by centrifugation (5 min at 14,000 rpm). Genomic DNA was prepared with a PureLink genomic DNA minikit (Invitrogen, Thermo Scientific). One microgram of genomic DNA was sent to GenOne Soluções em Biotecnologia (Rio de Janeiro, Brazil). Default parameters were used for all software unless otherwise specified. Short inserts of genomic DNA libraries were prepared using the NEBNext Ultra II DNA library prep kit for Illumina (New England BioLabs, USA). One gigabyte of whole-genome sequencing (150-bp paired-end reads; Q30 > 80%) was performed on a NovaSeq 6000 system (Illumina), which yielded 1.2 Gb of raw data. The generated reads were evaluated for quality using FastQC v.0.11.9 (5). Bases with a score of less than Q15 were trimmed, and reads shorter than 50 nucleotides (nt) and their adaptors were removed using Trimmomatic v.0.39 (6).

*De novo* assembly was carried out using Unicycler v.0.4.8 (7) and CAP3 v.02/10/15 (8). Prokaryotic Genome Annotation Pipeline (PGAP) annotation detected a total of 5,536 genes and 5,440 coding sequences. The results are summarized in Table 1.

**TABLE 1:** Genome features and characterization of *B. paramycoides* LB_RP2

Parameter(s)	Data
Morphology and metabolism	Rod shaped, Gram positive, catalase (+), facultatively anaerobic^*a*^
No. of raw reads	7,699,810
No. of contigs	76
Draft genome size (Mb)	5.34
*N*_50_ (bp)	216,793
*L*_50_	8
GC content (%)	35.2
	
NCBI PGAP^*b*^ genome analysis annotation	
No. of genes (coding)	5,173
No. of CDS^*c*^	5,173
No. of rRNAs	8
No. of tRNAs	83
No. of pseudogenes	267

a Liu et al. (4).

b NCBI Prokaryotic Genome Annotation Pipeline (9, 10).

c CDS, coding sequences.

Whole-genome sequence analysis (tetra-nucleotide analysis [TNA] and average nucleotide identity [ANI] based on BLAST+ [ANIb] and MUMmer calculation [ANIm]) were performed using the JSpecies Web server (JSpecies WS) (11). The analysis revealed that LB_RP2 had an ANIb value of 97.77%, an ANIm value of 98.24%, and a TNA value of 0.99968 compared to *B. paramycoides* strain NH24A2, thus confirming that LB_RP2 is a Bacillus paramycoides strain.

A cluster of genes in the poly(3-hydroxybutyrate) [P(3HB)] and poly(3-hydroxybutyrate-3-hydroxyvalerate) [P(3HB-co-3HV)] biosynthesis pathway, *phaA*, *phaP*, *phaQ*, *phaR*, *phaB*, and *phaC*, were identified in the genome. PhaC, with its subunit PhaR, is classified as a class IV PHA synthase and has a substrate specificity for 3-hydroxyacyl-coenzyme A (CoA) moieties, which leads to short-chain length PHA synthesis (12). Other important putative enzymes were found, such as acetate kinase and phosphate acetyltransferase, that permit the conversion of propanoate to propionyl-CoA, which is the precursor of 3-(*R*)-hydroxyvaleryl-CoA substrate and can be polymerized into a P(3HB-co-3HV) copolymer (13). These results suggest that *B. paramycoides* LB_RP2 can synthesize polyhydroxyalkanoates, which is a class of polymers that has great industrial potential due to its high applicability.

### Data availability.

The full sequence assembly is available from the genome database at GenBank, under accession number JACAXA010000018.1 (BioProject accession number PRJNA641466). The SRA accession number is SRR14323286.
