# Correction for de Castro et al., “Draft Whole-Genome Sequence of *Bacillus paramycoides* LB_RP2, a Putative Polyhydroxyalkanoate-Producing Bacterium Isolated from an Amazonian Blackwater River”

**DOI:** 10.1128/mra.00666-24

**Published:** 2024-07-17

**Authors:** Lorena Mota de Castro, Choon Pin Foong, Mieko Higuchi-Takeuchi, Eraldo Ferreira Lopes, Keiji Numata, Suelen Dias da Silva, Luana da Silva Nonato, Adolfo José da Mota, José Odair Pereira

## AUTHOR CORRECTION

Volume 10, no. 30, e00438-21, 2021, https://doi.org/10.1128/mra.00438-21. Page 2, Acknowledgments: “…for A. J. da Mota” should read “…for A. J. da Mota and by Fundação de Amparo a Pesquisa do Estado do Amazonas (FAPEAM) (POSGRAD 2020).”

